# *Dryas* as a Model for Studying the Root Symbioses of the Rosaceae

**DOI:** 10.3389/fpls.2019.00661

**Published:** 2019-06-04

**Authors:** Benjamin Billault-Penneteau, Aline Sandré, Jessica Folgmann, Martin Parniske, Katharina Pawlowski

**Affiliations:** ^1^Institute of Genetics, Faculty of Biology, LMU Munich, Martinsried, Germany; ^2^Department of Ecology, Environment and Plant Sciences, Stockholm University, Stockholm, Sweden

**Keywords:** *Dryas*, model-plant, *Dryas drummondii*, *Dryas octopetala*, Rosaceae, genome comparison

## Abstract

The nitrogen-fixing root nodule symbiosis is restricted to four plant orders: Fabales (legumes), Fagales, Cucurbitales and Rosales (Elaeagnaceae, Rhamnaceae, and Rosaceae). Interestingly all of the Rosaceae genera confirmed to contain nodulating species (i.e., *Cercocarpus*, *Chamaebatia*, *Dryas*, and *Purshia*) belong to a single subfamily, the Dryadoideae. The *Dryas* genus is particularly interesting from an evolutionary perspective because it contains closely related nodulating (*Dryas drummondii)* and non-nodulating species (*Dryas octopetala)*. The close phylogenetic relationship between these two species makes *Dryas* an ideal model genus to study the genetic basis of nodulation by whole genome comparison and classical genetics. Therefore, we established methods for plant cultivation, transformation and DNA extraction for these species. We optimized seed surface sterilization and germination methods and tested growth protocols ranging from pots and Petri dishes to a hydroponic system. Transgenic hairy roots were obtained by adapting *Agrobacterium rhizogenes*-based transformation protocols for *Dryas* species. We compared several DNA extraction protocols for their suitability for subsequent molecular biological analysis. Using CTAB extraction, reproducible PCRs could be performed, but CsCl gradient purification was essential to obtain DNA in sufficient purity for high quality *de novo* genome sequencing of both *Dryas* species. Altogether, we established a basic toolkit for the culture, transient transformation and genetic analysis of *Dryas* sp.

## Introduction

Nitrogen and phosphate are key nutrients for plant growth, but their availability is limited, especially in alkaline and calcareous soils (Vitousek et al., 2010; Lambers et al., 2012). Plants, with their limited capacity to retrieve nutrients from the soil, profit from interactions with beneficial microorganisms. Root endosymbiosis with arbuscular mycorrhizal fungi or nitrogen fixing bacteria are examples of this kind of interaction, where the microsymbiont is accommodated within root cells, leading to a gain of function that enables the plant to survive or even thrive in previous uninhabitable environments. Among terrestrial plants, the vast majority (80%) is able to develop arbuscular mycorrhiza (AM), a symbiosis with fungi of the Glomeromycotan (Delaux et al., 2013); one of the main benefits of the AM symbiosis is the ability of the fungi to improve the host plants’ access to phosphate. Despite the advantages of this symbiosis, some plant lineages have lost genes essential for its establishment. One such lineage is the Brassicaceae family that comprises the model plant *Arabidopsis* *thaliana* (Cosme et al., 2018). Another symbiotic system evolved for the acquisition of nitrogen through the cooperation with nitrogen fixing *Frankia* or rhizobia bacteria, the nitrogen-fixing root nodule symbiosis (RNS). The symbiosis is named after the specialized root organs formed by the plant, root nodules, which provide physiological conditions for the bacteria to fix atmospheric nitrogen and export a product of nitrogen fixation to the host plant. This endosymbiosis is restricted to plant species in the related orders Fabales, Fagales, Cucurbitales and Rosales, forming the FaFaCuRo clade (Soltis et al., 1995; Werner et al., 2014; Griesmann et al., 2018). Legumes (Fabales) and *Parasponia* sp. (Cannabaceae, Rosales) interact with rhizobia, while all other RNS forming plant species interact with the actinobacterium *Frankia* and consequently are called actinorhizal plants.

Genetic dissection of rhizobial symbiosis, mainly two model legumes – *Medicago* *truncatula* (Barrel medic) and *Lotus corniculatus* L. (bird’s-foot trefoil) *var. japonicus*, – has revealed symbiosis-related genes that are essential for nodule organogenesis, bacterial infection, and nitrogen fixation (see, e.g., Geurts et al., 2016). Recent phylogenomic studies have revealed that although most current members of the FaFaCuRo clade cannot form root nodules, the common ancestor of the FaFaCuRo clade was able to enter a symbiosis (Griesmann et al., 2018; van Velzen et al., 2018). However, it is not clear whether this ancestral symbiosis involved the formation of root nodules (Parniske, 2018). Due to their common origin, several similarities exist between actinorhizal and rhizobial symbioses, and the transfer of knowledge from legumes to actinorhizal plants improves our understanding of the main processes underlying the symbiosis with *Frankia* bacteria. Nevertheless, important aspects of actinorhizal symbioses remain unknown (Pawlowski and Bisseling, 1996; Perrine-Walker et al., 2011; Van Nguyen and Pawlowski, 2017). Consequently, research on actinorhizal plants could allow us to better understand the evolution of divergent symbiotic processes in RNS.

The Rosaceae family, belonging to the order Rosales, is globally the 4th most important plant family in terms of economic value (Vallée et al., 2016). Surprisingly, from *ca.* hundred Rosaceae genera only four, forming the basal subfamily Dryadoideae (Xiang et al., 2016), have been described to contain actinorhizal species that are able to enter a nitrogen-fixing RNS with *Frankia* bacteria (Pawlowski and Demchenko, 2012). The most basal genus of the Rosaceae, *Dryas*, is one of the most dominant dwarf shrubs among the arctic plant genera in terms of biomass. The taxonomy within the *Dryas* genus is controversial due to the existence of hybrids that occur naturally in the wild (Packer, 1994; Philipp and Siegismund, 2003). In areas where different *Dryas* species cohabit, natural hybrids were described between *Dryas integrifolia* and *Dryas octopetala* (Philipp and Siegismund, 2003), or between *Dryas. drummondii* and *D. integrifolia* (known as *D. x wyssiana*). The German botanist Franz Sündermann also created *D. x suendermannii* by crossing *D. drummondii* with *D. octopetala* (Packer, 1994), as part of the collection of the “Botantischen Alpengarten Sündermann” at Lindau, Germany, where the hybrids are maintained by clonal propagation.

Currently, three *Dryas* species, *D. drummondii*, *D. integrifolia*, and *D. octopetala*, are recognized; however, the genus is in need of taxonomic revision (Porslid, 1947; Böcher et al., 1968; Hultén, 1968; Yurtsev, 1997; Philipp and Siegismund, 2003; Skrede et al., 2006). The genus *Dryas* is unique in that it contains closely related nodulating and non-nodulating species which makes it an ideal model to study the evolution of root symbioses. Nodulation was reported for the first time in 1967 (Lawrence et al., 1967) in the North American species *D. drummondii* (Newcomb, 1981; Kohls et al., 1994; Figure 3A). The other species appear to be non-nodulating (Becking, 1970; Markham, 2009), but all *Dryas* species form ectomycorrhiza (Melville et al., 1988; Ryberg et al., 2009; Bjorbækmo et al., 2010; Botnen et al., 2014). The only exception may be *D. drummondii* because it has been described as ectomycorrhizal only on one occasion (Fitter and Parsons, 1987). The question whether this species can form ectomycorrhiza requires further examination.

The arctic-alpine species *D. octopetala* has a particularly wide distribution; it can be used for mapping refugial isolation and postglacial expansion during the glaciation in the Pleistocene in northern Europe (Philipp and Siegismund, 2003; Skrede et al., 2006). The plants typically grow in alkaline calcareous soils (Crocker and Major, 1955) and thus face nutritional limitations especially in terms of nitrogen and phosphorus (Lambers et al., 2012). The presence and abundance of *Dryas* species across all arctic and alpine tundra’s makes this genus a key player in arctic phylo- and bio-geography (Tremblay and Schoen, 1999; Skrede et al., 2006), landscape ecology (Eichel et al., 2016; Eichel et al., 2017) and mycology community ecology (Väre et al., 1992; Ryberg et al., 2009; Bjorbækmo et al., 2010; Brunner et al., 2017). The genus is of particular importance in research on climate change (see, e.g., McGraw et al., 2014; Gillespie et al., 2016; Panchen and Gorelick, 2017). Therefore, *Dryas* species also are integral to Citizen Science Projects, e.g., the Spatial Food Web Ecology Group of the University of Helsinki use *Dryas* sp. as base of two Ecosystem Ecology projects, the Arctic Parasitoid Project and the Global *Dryas* Project^1^, and the Climate Impact Research Centre in Abisko also includes *Dryas octopetala* in their target plants^2^.

*Dryas* species are diploid with an estimated haploid genome size of 250 Mbp (Griesmann et al., 2018) distributed over nine chromosomes (Potter et al., 2007), less than *Malus* ×*domestica* (apple) which is diploid or triploid with 750 Mbp, or *Rosa* which is tetraploid or triploid with 600 Mbp (Jung et al., 2013). The genome sequence of *D. drummondii* obtained from DNA purified with the protocol described here is publicly available; all data have been deposited in GigaDB (Griesmann et al., 2018). This small genome, combined with a generation time of less than a year, makes *Dryas* suitable as model genus for the Rosaceae family.

In this study, we focused on *D. drummondii* and *D. octopetala*. We omitted *D. integrifolia* because of its high similarity with *D. octopetala* (Skrede et al., 2006), the latter being more accessible and better researched. The close relation between *D. drummondii* and *D. octopetala* allows genomic comparisons in order to identify genes specifically involved in plant root endosymbiosis. We present the advances achieved in the development and adaptation of protocols in order to use *Dryas* as a model genus in Rosaceae research as well as to study the evolution of root symbioses.

## Materials and Methods

### *Dryas* Seeds and Ecotypes

Seeds of *D. drummondii* DA462 and *D. octopetala* DA460 were purchased from the seed producer Jelitto (Schwarmstedt, Germany). The Nymphenburg Botanical Garden of Munich supplied seeds for *D. drummondii* BGM. Ecotypes Albe. (origin Clearwater County, Alberta, Canada, collected in 2000); and Alas. (origin Alaska, United States, collected in 2002) were found in and supplied by the KEW Millennium Seed Bank (Royal Botanic Gardens, Kew, London, United Kingdom). *D. octopetala* ecotype E548 was harvested in the Italian Alps (approximate GPS coordinates: 46°24′36.7″N 11°37′48.2″E) by Anna Heuberger.

### Primers

Primers were designed based on the first draft genome of *D. drummondii* (Griesmann et al., 2018). They are as follows: GADPH forward 5′-CCCCAGTACGAATGCTCCCATGTTTG-3′, GADPH reverse 5′-TTAGCCAAAGGAGCAAGACAGTTGGTGG-3′; EF1-a forward 5′-TGGGTTTGAGGGTGACAACATGA-3′; EF1-a reverse 5′-GTACACATCCTGAAGTGGGAGACGGAGG-3′; 26S rRNA forward 5′-TACTGCAGGTCGGCAATCGG-3′, 26S rRNA reverse 5′-TCATCGCGCTTGGTTGAAAA-3′. ITS primers were designed based on Cheng et al. (2016): ITS forward 5′-CCTTATCAYTTAGAGGAAGGAG-3′, ITS reverse 5′-RGTTTCTTTTCCTCCGCTTA-3′.

### Seed Storage and Sterilization

Based on advice from seed producers and on results from Nichols (1934) who observed that without prior refrigeration, germination of several alpine species was considerably reduced, we assumed that seeds of *Dryas* species might require cold stratification prior to germination and therefore stored them at 4°C. *Dryas* seeds were surface sterilized by immersion in 30% H_2_O_2_ (10 min for *D. octopetala*; 15 min for *D. drummondii*) and washed three times with sterile H_2_O. These experiments were performed with four biological replicates per species, with at least 100 seeds per replicate.

### Growth Systems

Sterilized seeds were transferred on 1% agar-water plates and incubated in the dark at 22°C for 12 and 8 days for *D. octopetala* and *D. drummondii*, respectively. Several sources of agar were tested such as Bacto^TM^ agar (Becton Dickinson and company) and agar Kalys HP 696 (Kalys SA, Bernin, France). The germination assays were set up in the dark because this reportedly increased germination rates Bliss (1958). After germination, seedlings of *Dryas* spp. were grown on plates, in a hydroponic system or in pots.

Growth on plates was performed on ¼ Hoagland’s pH 5.8 (using the protocol for N-free medium; Hoagland and Arnon, 1950) and adding 1 KNO_3_ to a final concentration of 1 mM) with 0.4% of Gelrite (Duchefa, Haarlem, Netherlands), at 22°C, 55% of humidity with 16 h-light/8 h-dark cycles. After 1 week, plantlets were transferred either into pots or Weck jars (containing production substrate A210; Stender AG, Germany) or into a hydroponic system.

The hydroponic system consisted of a standard 1 mL pipette tip box in two parts: the bottom part contained 250 mL of growth medium (¼ Hoagland, 1 mM KNO_3_, pH 5.8) and the tip holder with 24 holes in which the plantlets were inserted (Figure 2). To avoid seedlings or young plantlets falling into the medium compartment, the holes were covered with adhesive tape and plantlets were introduced through thin slits cut into the tape. The growth medium was changed twice per week. The hydroponic system was kept in a growth cabinet at 22°C, 55% humidity with 16 h-light/8 h-dark cycles for a maximum of 4 months.

Plants in pots were transferred to the greenhouse (day temperature 21–24°C, night temperature 18–21°C, with additional lighting from 6:00 to 10:00 h and from 15:00 to 22:00 h). The pots were filled either with sand:vermiculite (2:1) or with propagating substrate (A210 Stender AG, Germany). Note that temperature and light conditions were applied as available in our plant growth facilities and not experimentally optimized for *Dryas*.

### Cutting Propagation

For clonal propagation, young and soft shoots of *Dryas* spp. were cut after the third internode (2–5 cm) above the woody part of the shoot. These explants were directly transferred into moist production substrate A210 (Stender AG, Germany), then kept under plastic cover in the greenhouse. High humidity was maintained under the cover by spraying with water every 2 days for 2 weeks; thereafter, spraying was stopped and cuttings were kept in moist soil under the cover until new leaves had developed and the covers were removed. Three series of ca. 20 cuttings per species were cultivated in the greenhouse during different seasons.

### Hairy Root Transformation

We established a protocol for hairy root transformation in *Dryas* spp. by adapting *Lotus* protocols. The *Agrobacterium rhizogenes* strain AR1193 (Stougaard et al., 1987) was used because it had been shown to be very efficient for some plant species such as pea (Clemow et al., 2011) and because it was one of the strains available in our lab previously successfully tested for *Lotus japonicus* hairy root transformation.

*Agrobacterium rhizogenes* AR1193 bacteria carrying a Golden Gate LIIIβ F A-B (Binder et al., 2014) plasmid containing the *mCherry* gene under control of the *Ubiquitin* promoter (*AtUbi10*pro) as transformation marker (Pimprikar et al., 2016), were grown in liquid culture (LB medium with 50 μg mL^-1^ each of rifampicin, carbenicillin and kanamycin) at 28°C overnight. Bacteria were collected via a centrifugation step (15 min at 4.369 ×*g*) and resuspended in water to obtain the wanted OD_600_ (0.01; 0.1; 1; 7.2). Cut hypocotyls of 10–12 days old axenically grown *Dryas* spp. seedlings were dipped in the bacteria suspension and placed on ¼ Hoagland (1 mM KNO_3_, pH 5.8), 0.4% Gelrite (Duchefa, Haarlem, The Netherlands) plates. The plates were kept for 4 days in the dark at 22°C, then under a 16 h-light/8 h-dark cycle with 55% humidity. To prevent overgrowth of bacteria and dehydration, the plants were transferred onto new plates every week. Four to six weeks after transformation, roots were screened using a Leica MZ16 FA stereomicroscope (Leica Microsystems GmbH, Wetzlar, Germany) using the N3 filter from Leica (BP 546/12;600/40).

### DNA Extraction and PCR Reactions

The CsCl gradient DNA extraction method was performed according to Ribeiro et al. (1995). Six to ten grams of leaves (mix of young and old from the same plant) were ground by hand using pistil and mortar with 4 g of PolyclarAT in liquid nitrogen. For the other extraction methods, the two youngest leaves of a shoot with the apical meristem were used as starting material. After being shock frozen in liquid nitrogen, they were ground with a Retsch Mill MM400 (Fa. Retsch, Haan, Germany) two times at 30 Hz for 30 s in 2 mL Eppendorf tubes containing 2 mm diameter stainless steel beads each. The “classical CTAB” extraction method is described in Doyle and Doyle (1987), whereas the “PVP/NaCl” extraction method was developed by Khanuja et al. (1999) based on the classical CTAB method.

PCRs were performed on 1 μl of DNA (20–700 ng of DNA, usually ca. 100 ng) using GoTaq^®^ DNA polymerase (Promega, Germany), SYBR Green buffer and 0.2 μM of each primer. Amplifications were carried for 5 min at 95°C, followed by 35 cycles (30 s at 95°C, 30 s at 60°C, and 40 s at 72°C), and a final extension for 1 min at 72°C. Electrophoresis of a 4 μL of PCR reaction was performed on a 3% agarose gel for 100 min at 130 V. DNA was visualized with UVP UV solo touch from Analytik Jena© (Jena, Germany) after incubation of the gel for 10 min in an Ethidium bromide bath at 2 ng mL^-1^.

### RNA Extraction

Leaves, seedlings and root systems were shock frozen in liquid nitrogen. RNA of ground material (with the same procedure previously described for DNA extraction) was extracted using the Spectrum^TM^ Plant Total RNA Kit (Sigma-Aldrich, CA, United States) without adaptation in the protocol except for older and thicker leaves for extraction of which Polyclar AT was added. The RNA was treated with DNAse (Invitrogen^TM^ TURBO DNA-free Kit; Carlsbad, CA, United States) and tested for purity and integrity with a Bioanalyzer from Agilent (Agilent Technologies, Palo Alto, CA, United States). Twelve independently isolated RNA samples were analyzed.

## Results and Discussion

To establish *Dryas* as new model genus in the laboratory, we developed cultivation protocols under controlled conditions.

### *Dryas* Seeds and Germination

**FIGURE 1:**
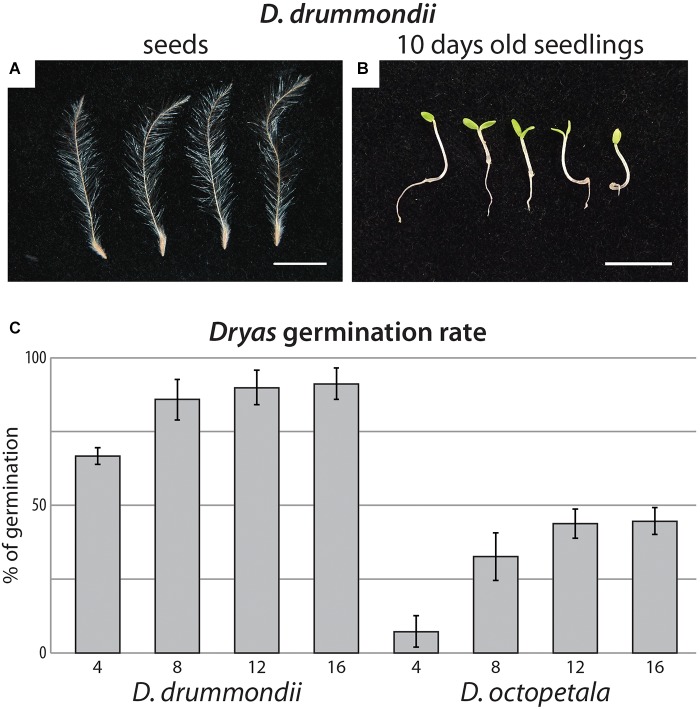
*Dryas* spp. from seeds to seedlings. **(A)** Isolated anemochorous silky-feathery achenes from *Dryas drummondii*. **(B)** 10-day-old seedlings of *D. drummondii*. Scale bars denote 1 cm. **(C)** Time course of *Dryas* sp. germination after seed surface sterilization. Displayed are means (*n* = 4 biological replicates with at least 100 seeds per replicate) and standard errors.

Fungal contamination of seeds was often observed, whether they were collected in the field or obtained from a professional seed producer. In our study, white fungal hyphae growing out of the seeds led to seedling death at an early stage. *Dryas* seeds were quite sensitive to different surface sterilization procedures: any traces of ethanol would completely inhibit germination, while the thinness of the seed coat rendered the use of sulphuric acid for scarification risky. Furthermore, the contaminating fungi were quite resistant to NaOCl. However, after stratification of *Dryas* seeds at 4°C (Figure 1A), most efficient sterilization and highest germination rates were observed using hydrogen peroxide. Indeed approximately 100% of *D. octopetala* seeds and between 99 and 100% of *D. drummondii* seeds were free of contaminants after the procedure. Four replicates, with at least 100 seeds per replicate, were observed every 2 days. For *D. drummondii*, the maximal germination rate (85%) was obtained 8 days post-sterilization, whereas the maximum germination rate of *D. octopetala*, *ca.* 40%, was only reached 12 days post-sterilization (Figure 1C).

The time difference of 4 days to reach maximum germination observed between *D. drummondii* and *Dryas octopetala* was consistently observed at least within the samples tested: seeds of all *D. drummondii* seed sources examined germinated within 8 days and *D. octopetala* seeds from all sources available within 12 days. This phenotype was consistent not only for seeds produced in the greenhouse or botanical garden during the same month, but also for commercial seeds, the age of which was unknown. However, given that *D. octopetala* has a very wide distribution, it is possible that the two seed sources examined do not encompass the entire variability of the species.

### *Dryas* Growth Systems

We examined different growth conditions and systems including Gelrite and agar plates, hydroponic systems and classical pots. These distinct growth systems combine diverse advantages for research such as axenic culture, conditions for root system observations and for inoculation with the microsymbiont. *Frankia* strains able to nodulate *D. drummondii* have not yet been successfully cultured (Normand et al., 2017), necessitating infection with crushed nodules. As these nodules carry a rich fungal and bacterial microbiome on the surface, inoculation of *Dryas* with these nodules while maintaining a gnotobiotic system is challenging. On the other hand, plants grown in pots do not represent the most suitable system for root analyses. The process of cleaning the soil from the roots stresses the plant and furthermore, the harvesting of root systems entails the risk of breaking thin and fragile lateral roots and root hairs. To circumvent this drawback, plants can be grown in Petri dishes. For *Dryas* species this system was suited for early stages of development; for experiments exceeding 4–5 weeks, root and shoot growth required more space. Furthermore, shielding of plates never totally protected the roots from light, and a long exposure of roots to light tends to interfere with the analysis of root responses to any treatment. Exposure of roots to direct light modifies their transcriptome (Hemm et al., 2004) and often leads to stress responses, which can perturb the analyses and cause misleading effects. Hydroponic systems offer the possibility to observe the roots in a non-invasive way while also shielding them from light. They can be used with or without an inert substrate that mimics physical soil contact. The fact that *Dryas* species can grow in well-aerated soil but can also tolerate flood periods (West et al., 1993), suggested the use of hydroponics as a method of choice.

Once germinated and grown on 1% agar plates with classical plant media like B5 or MS (Murashige and Skoog, 1962; Gamborg et al., 1968; Duchefa, Haarlem, Netherlands) or Fåhræus medium (Fåhraeus, 1957) with 1 mM KNO_3_, *Dryas* species seedlings turned reddish, likely due to μlanin production, a response typically interpreted as stress- or defense-related. This anthocyanin production was less pronounced when the seedlings were grown on 0.4% Gelrite with ¼ strength Hoagland medium containing 1 mM KNO_3_ (Figure 1B). Moreover, after 2 weeks on plates, *Dryas* spp. plantlets grown on ¼ strength Hoagland medium showed darker green cotyledons and further developed root systems than on B5 medium. Thus, among the tested media for *Dryas* seedlings on gel-forming media, the best results were obtained with 0.4% Gelrite containing ¼ strength Hoagland solution.

**FIGURE 2:**
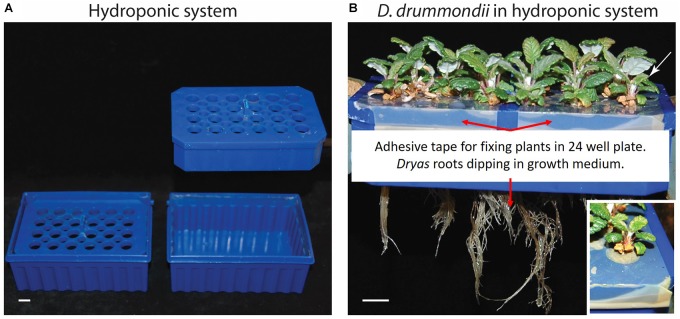
Hydroponic system for *Dryas* spp. **(A)** Overview of the hydroponic system: assembled (left) and split up (right). **(B)** Hydroponic culture of *Dryas drummondii* after 7 weeks in the hydroponic system; the white rectangle shows a close-up view of the plant labeled with a white arrow. Scale bars denote 1 cm.

After germination on plates, *Dryas* species. plantlets were transferred to a hydroponic system (Figure 2A) with ¼ Hoagland solution. In this system *Dryas* species plants grew and developed without obvious stress symptoms like accumulation of anthocyanins; they formed well-developed primary and lateral roots, and the speed of shoot development resembled that of pot-grown plants (Figure 2B). Altogether, *Dryas* species plants adapted very well to the hydroponic system tested. The absence of gel and soil substrates offers the opportunity to perform non-invasive observations of roots.

### Sexual Propagation of *Dryas* Species

*Dryas* is a perennial plant genus. *D. drummondii* has been found to flower in its fifth year (Lawrence et al., 1967), indicating a long generation time that renders crossing experiments difficult. However, nodulation, plant growth and flowering processes in *Dryas* sp. seem to be extremely dependent on the environment and on light quality and intensity (Kohls et al., 1994). Therefore, we attempted to reduce the generation time under greenhouse conditions.

**FIGURE 3:**
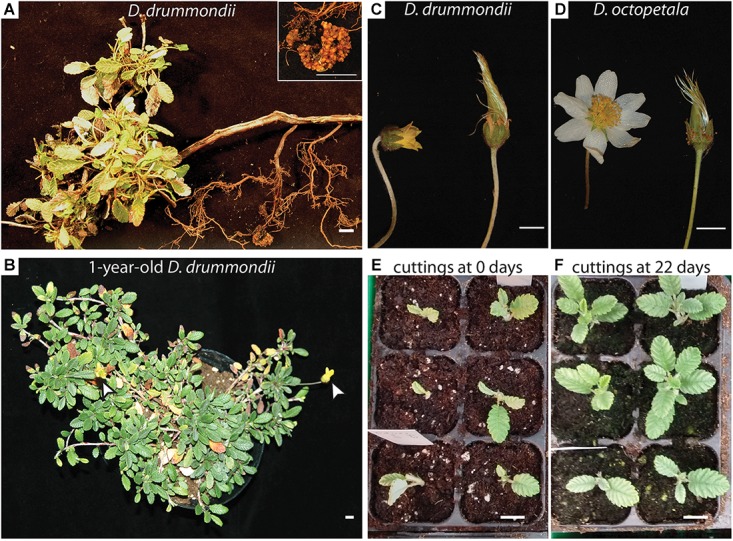
Sexual and vegetative propagation of *Dryas* spp. **(A)** Part of a *Dryas drummondii* plant with a dense leaf rosette and lignified roots, coming from a woody shoot, bearing a nodule (shown magnified in the inset). The plant was collected in the Nymphenburg Botanical Garden of Munich. **(B)** 1-year-old *D. drummondii* plant flowering in the greenhouse under a higher-pressure sodium lamp (white arrows indicate flowers). **(C–D)** Flowers of *D. drummondii* **(C)** and *D. octopetala* **(D)** in full bloom and in fructification. **(E,F)** Cuttings of *D. drummondii* at day 0 **(E)** and after 22 days of growth **(F)**. Scale bars denote 1 cm.

In our study, flower and seed production did not occur when plants were grown at a distance of 2 m from standard high-pressure mercury vapor lamps (providing 90 μmol m^-2^ s^-1^ at the plant level) used in initial trials (fluorescent lamps were not tried for flowering as seedlings from both species were growing significantly more slowly under them than under mercury vapor lamps for the first 5 weeks after germination). However, when the plants were placed at a distance of 2 m under high-pressure sodium vapor lamps (150 μmol m^-2^ s^-1^) for 16 h per day, flowering was induced (Figure 3B–D). High-pressure sodium lamps provide light with a richer emission in yellow-orange and a red/far red ratio shifted to the far red compared to standard high-pressure mercury vapor lamps and fluorescent lamps. The flowers produced seeds (Figure 1A) within less than a year after germination, whether the plants originated from cuttings or from sexual propagation. This is a far shorter generation time than the 5 years required for *D. drummondii* according to Lawrence et al. (1967). In the field, *Dryas* spp. flower primordia are formed during the summer, i.e., far in advance of flowering, which occurs in the next year shortly after snowmelt, with most individuals flowering within a month (Lawrence et al., 1967). This behavior suggests that the development of floral primordia and blooming depends on photoperiod or vernalization (or both). It is surprising that light from high-pressure sodium lamps, characterized by a lower red/far red ratio, leads to induction of flowering in an arctic/alpine species like *D. octopetala*, and of a species that has been described as extremely shade-sensitive *(D. drummondii*; Cooper, 1931). We observed that plants grew much better outdoors than in the glasshouse, but did not identify the limiting parameters. At any rate, changes of light period and temperature might further speed up the induction of flowering and shorten the generation time.

Seed set under greenhouse conditions occurred at ca. 75% of all *D. drummondii* flowers and at ca. 65% of all *D. octopetala* flowers. In their natural habitat, *Dryas* species combine autogamy and allogamy; seed set is improved when insects are available for pollination (Kevan, 1975; Roslin et al., 2013; Tiusanen et al., 2016, 2019). In the greenhouse, while some insects were usually present, seeds would be formed by flowers covered with paper bags, indicating that all seed batches used gave rise to plants that could perform self-fertilization.

Manual pollination was performed in the greenhouse and in a botanical garden to obtain hybrids. While the large and open flowers of *D. octopetala* (Figure 3D) made manual pollination possible, *D. drummondii* flowers were never completely open during full bloom (Figure 3C). Therefore, crossings of female *D. octopetala* with male *D. drummondii* were attempted. However, these attempts were not successful. Flowers of species contain multiple stamens, and often, after manual pollination it turned out that not all of them had been removed.

Altogether, sexual reproduction of *Dryas* spp. was feasible in a laboratory context when sufficient light intensity of a suitable spectrum was provided.

### Clonal Propagation of *Dryas* spp.

Given that *Dryas* spp. are partially allogamous and experimental studies require homogenous plant material, the use of *Dryas* as a model genus requires an easy protocol for vegetative propagation. In the wild, clonal growth of *Dryas* species enables individuals to persist and grow in extreme environments where sexual proliferation is often unsuccessful (Wookey et al., 1995), and where individual clones of *D. octopetala* commonly live for more than 100 years (Kihlman, 1890; Crawford, 1989). Thus, clonal propagation of *Dryas* species was expected to be easy. For clonal propagation by cuttings, three series of ca. 20 cuttings per species were grown in a greenhouse at different times of the year. Two to three cm of *Dryas* stems containing one node were transferred into moist soil (Figure 3E) in a small growth container with a transparent plastic lid for conservation of high humidity levels. Under these conditions, 65–95% of the *Dryas* cuttings developed roots within 3 weeks in the absence of hormonal treatments (Figure 3F). Once the shoots had successfully rooted, the plants were transferred into single pots and grown under standard greenhouse conditions. This easy protocol for vegetative propagation of *Dryas* species by cuttings in the glasshouse represents an important tool for performing experiments on a high number of plants that have the same genotype, and it obviates the requirement for seeds.

### Hairy Root Transformation of *Dryas* spp.

For a model plant, a protocol for genetic modification is important in order to analyze the expression of marker gene promoter-reporter gene fusions, or to perform reverse genetics. Hairy root transformation mediated by *Agrobacterium rhizogenes* is the most commonly used technique to introduce chimeric constructs into plant roots. The fact that this method does not transform the shoot is no hindrance to the study of root symbioses; *A. rhizogenes*-mediated hairy root transformation is routinely used not only in the model legumes *Lotus corniculatus* var. *japonicus* (Díaz et al., 2005) and *Medicago truncatula* (Boisson-Dernier et al., 2001) but also for actinorhizal plants like *Datisca glomerata* (Markmann et al., 2008), *Casuarina glauca* (Diouf et al., 1995) and (Imanishi et al., 2011) and non-FaFaCuRo plants like tomato (Ron et al., 2014).

**FIGURE 4:**
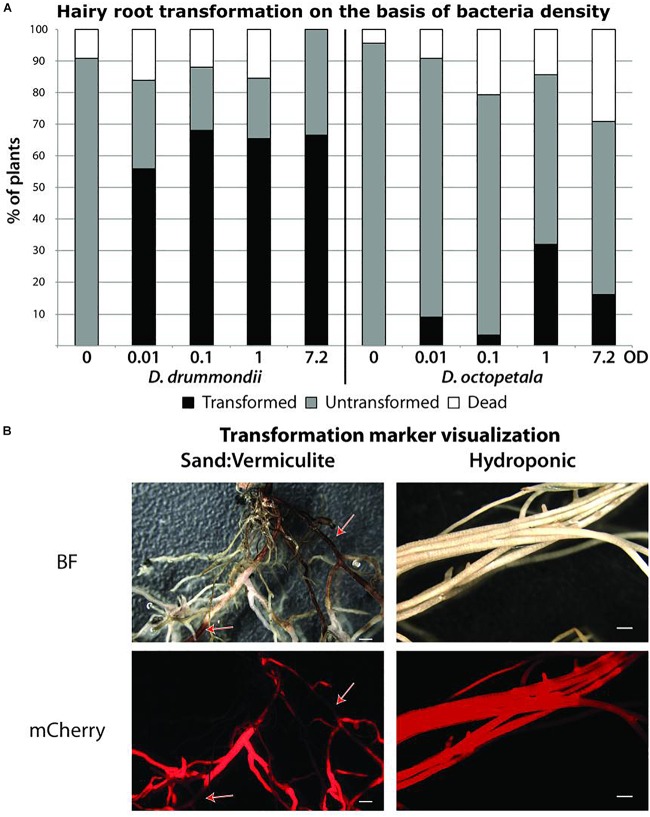
*Agrobacterium rhizogenes-*mediated transformation of *Dryas* spp. **(A)** Success rates of hairy root transformation of *D. drummondii* and *D. octopetala* depended on the bacteria density. Percentages of dead (white boxes), surviving untransformed (gray boxes) and transformed (black boxes) root systems were determined 5 weeks after transformation on plants grown in Petri dishes. Transformation was determined based on mCherry fluorescence. **(B)** Visualization of the mCherry transformation marker of *D. drummondii* hairy roots after 7 weeks of growth in Weck Jars containing sand:vermiculite (left panel) vs. growth in the hydroponic system (right panel). Red arrows point at lignified part of the roots. BF = bright field; mCherry = mCherry fluorescence. Scale bars denote 1 mm.

In order to develop a hairy root transformation protocol, we inoculated axenically grown *Dryas* spp. seedlings with *A. rhizogenes* at different cell densities. 5 weeks after transformation, the composite plants on plate were evaluated. For *D. drummondii*, a transformation efficiency of 55–70% was obtained under all conditions tested, while *D. octopetala* plants died more frequently in response to infection with *A. rhizogenes*. The use of higher bacterial densities had a negative effect on plant survival, while lower bacterial densities reduced transformation efficiency. Here, the best compromise between low mortality and transformation rate for *D. octopetala* was observed when the *A. rhizogenes* suspension was adjusted to an OD_600_ of 1. However, the transformation efficiency was still low with only 30% (Figure 4A). The experiment was repeated three times using an *A. rhizogenes* suspension adjusted to an OD_600_ of 1 on ca. 70 seedlings per species. In all cases, the results were the same: 55–70% transformation for *D. drummondii*, maximally 30% transformation for *D. octopetala*.

Previous studies have shown that hairy roots induced by different bacterial strains can vary in morphology and production of secondary metabolites (Thwe et al., 2016); it was also shown that plant defense reactions, phytohormone signaling and secondary metabolism could be affected by high expression levels of the agrobacterial *rolB* gene (Bulgakov et al., 2018). Thus, the difference in the reactions of two closely related species to the same *A. rhizogenes* strain is interesting. At any rate, since only one *A. rhizogenes* strain was used in this study, the use of other strains might leave room for further optimization of hairy root transformation of *D. octopetala*.

Up to 7 weeks after transfer to pots or to the hydroponic system, transgenic roots showed healthy growth and expressed the transformation marker mCherry driven by the ubiquitin promoter (Figure 4B). However, roots growing in particle substrates such as sand:vermiculite, developed sections with increased lignification, hence more autofluorescence and opacity. This led to the quenching of the mCherry signal as highlighted by the red arrows in Figure 4B. In contrast, when plants were grown in the hydroponic system, lignification was less pronounced. Thus, the hydroponic system is well suitable for the observation of fluorescent proteins in *Dryas* spp. hairy roots.

The ability to clone *Dryas* spp. genes combined with the capacity to introduce chimeric constructs into root systems opens the possibility to study *Dryas* genetics in depth, allowing cross-species complementation, as well as transient expression, protein localization, and reverse genetics using CRISPR/Cas or RNAi methods.

### Nucleic Acid Extraction From *Dryas*

For molecular biological studies, DNA and RNA have to be isolated with high purity, integrity and yield to be used for sequencing or reverse transcription, respectively. This was particularly challenging since the woody nature of *Dryas* species and the composition of the leaves adapted to harsh environmental conditions led to the presence of contaminants interfering with nucleic acid extraction protocols.

**FIGURE 5:**
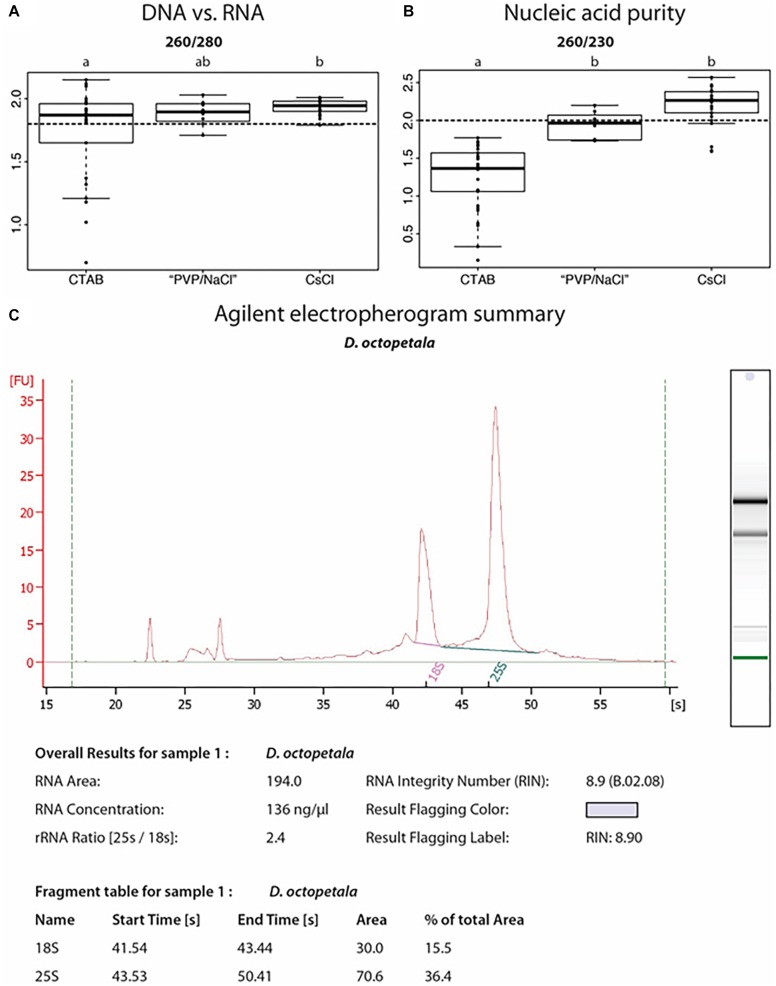
Nucleic acid extractions from *Dryas* spp. **(A,B)** Ratios of UV absorbance at 260 nm vs. 280 or 230 nm, from DNA samples isolated from *Dryas drummondii* and *Dryas octopeatala* using three different methods: the classical CTAB method = “CTAB”; an adapted CTAB method for difficult plants = “PVP/NaCl” and a method involving a Caesium chloride gradient centrifugation = “CsCl.” An OD_260_/OD_280_ ratio **(A)** for nucleic acids vs. protein of at least 1.8 (dashed line) is generally accepted as denoting “pure DNA.” OD_260_/OD_230_ **(B)** values for nucleic acids vs. polysaccharides should be higher than 2.0 (dashed line; Green and Sambrook, 2012). All DNA isolations were performed on 30 biological replicates per method. **(C)** Agilent Bioanalyzer electropherogram analysis of RNA isolated from *D. octopetala*, showing RNA integrity as determined by an RNA Integrity Number (RIN) of 8.9.

We tested different DNA extraction protocols on *D. drummondii* and *D. octopetala*, performing at least 30 extractions per method. DNA isolated from *Dryas* spp. with classical CTAB extraction protocols had an UV absorbance ratio at 260/280 of *ca.* 1.8, but the 260/230 ratio was always below 1.8, indicating polysaccharide contamination (Figure 5A,B). Several established DNA extraction methods were tested (Supplementary Table S1), but none of them led to a yield and purity sufficient for robust PCRs and *de novo* whole genome sequencing. However, a CTAB protocol adapted for recalcitrant plant material (Khanuja et al., 1999; “PVP/NaCl”) by addition of PVP, followed by a high salt lysis buffer and extraction with chloroform:isoamyl alcohol (24:1, v/v), resulted in good quality DNA suitable for PCRs with reproducible results (Figure 5B). Yet, the DNA yield and quality required for genome sequencing was so far only achieved using a modified Dellaporta et al. (1983) protocol followed by a CsCl gradient centrifugation as described by Ribeiro et al. (1995). The DNA extracted using this last method was used for *de novo* whole genome sequencing performed in collaboration with the Beijing Genomics Institute (BGI, China). The first version of the *D. drummondii* genome was used in a phylogenomic comparison study by Griesmann et al. (2018).

The Spectrum^TM^ Plant Total RNA Kit (Sigma-Aldrich) was used in order to extract RNA from different organs of *Dryas* spp. When Polyclar AT was added during the grinding step for recalcitrant samples (e.g., mature leaves and lignified roots), this method resulted in RNA of suitable integrity and purity for the performance of reverse transcription-quantitative PCR as indicated by the RNA integrity number (Figure 5C). RNA extracted from roots, leaves and seedlings following this method was used by the BGI in order to assist gene prediction for the *D. drummondii* genome (Griesmann et al., 2018). The transcripts were mapped to the protein-coding gene models, identified using the MAKER-P pipeline (version 2.31; Campbell et al., 2014), in order to obtain gene characteristics (size and number of exons/introns per gene, distribution of genes, features of splicing sites, etc.).

All method comparisons are summarized in Supplementary Table S1.

### PCR Amplification of *Dryas* spp. gDNA Fragments Using the *D. drummondii* Genome for Primer Design

**FIGURE 6:**
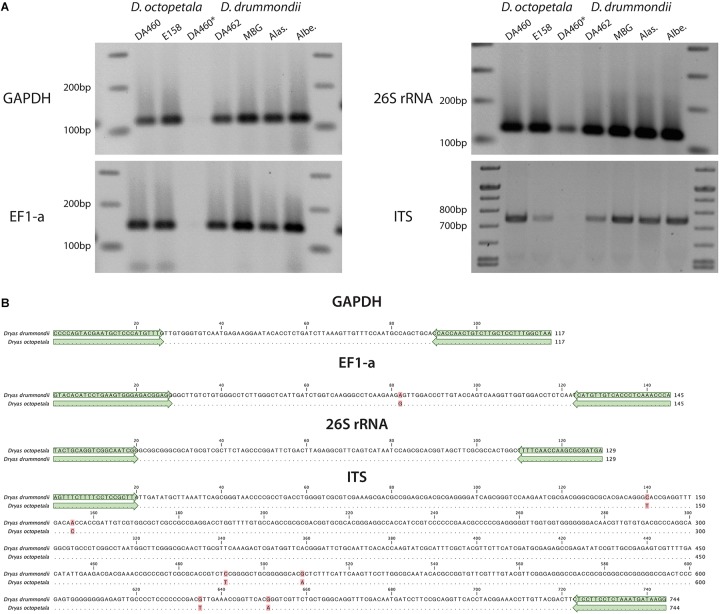
PCR of marker genes in *Dryas* spp. **(A)** PCR products were amplified from different marker genes (GADPH, EF1-a, 26S rRNA and ITS) using gDNA from *Dryas octopetala* ecotypes (“DA460” and “E548”) and *Dryas drummondii* ecotypes (“DA462,” “MBG,” “Alas.” and “Albe.”) extracted with the PVP/NaCl method (DA460^∗^ represents gDNA of *D. octopetala* ecotype DA460 extracted with the CTAB method). **(B)** Nucleotide alignment of the fragments from *D. drummondii* ecotype DA462 and *D. octopetala* ecotype DA460. Matching residues are marked as dots and differences are highlighted in red. Green arrows highlight the primers used.

We tested the suitability of the DNA preparations resulting from different protocols as templates for PCR. Based on the published *D. drummondii* genome (Griesmann et al., 2018). Primers were designed based on the first version of the *D. drummondii* genome. The targets were regions in the internal transcribed spacer (ITS) of nuclear ribosomal DNA, 26S ribosomal RNA (26S rRNA), glyceraldehyde 3-phosphate dehydrogenase (GAPDH) and the elongation factor 1-alpha (EF1-a). Using *D. octopetala* gDNA as template, fragments were amplified and sequenced as well. The amplification confirms that the DNA preparations were of sufficient quality, while high sequence conservation in these regions highlights the similarity between *D. drummondii* and *D. octopetala*. Indeed, the size of amplicons of both species were similar and their sequences presented few single nucleotide polymorphisms (Figure 6).

### *Dryas* as Model Genus for the Rosaceae

With the basic but indispensable procedures and protocols for cultivation, vegetative and sexual propagation, hairy root transformation of and nucleic acid isolation from *Dryas* spp. described in this study, *Dryas* emerges as a new model genus to study important traits associated with survival in arctic and alpine conditions, including the formation of root symbioses with bacteria and ectomycorrhizal fungi.

## Author Contributions

KP: proposal of *Dryas* as a promising model system to study root symbioses. MP, BB-P, and KP: conceptualization. BB-P, AS, and KP: methodology. JF: establishment of *Dryas* hairy root transformation. KP and BB-P: high quality nucleic acid extraction. BB-P and AS: visualization. BB-P: writing – original draft. BB-P, AS, MP, and KP: writing – review and editing. MP: funding acquisition. MP: supervision of AS and JF. KP and MP: supervision of BB-P.

## Conflict of Interest Statement

The authors declare that the research was conducted in the absence of any commercial or financial relationships that could be construed as a potential conflict of interest.

## Footnotes

1 https://www.helsinki.fi/en/researchgroups/spatial-food-web-ecology/research

2 https://www.arcticcirc.net/our-projects/
